# Variation in Length of Stay and Outcomes among Hospitalized Patients Attributable to Hospitals and Hospitalists

**DOI:** 10.1007/s11606-012-2255-6

**Published:** 2012-11-06

**Authors:** James S. Goodwin, Yu-Li Lin, Siddhartha Singh, Yong-Fang Kuo

**Affiliations:** 1 Department of Medicine and Sealy Center on Aging, University of Texas Medical Branch, Galveston, TX USA; 2 Department of Medicine, Medical College of Wisconsin, Milwaukee, WI USA

**Keywords:** hospitalist, length of stay, hospitalization, Medicare

## Abstract

**BACKGROUND:**

There have been no prior population-based studies of variation in performance of hospitalists.

**OBJECTIVE:**

To measure the variation in performance of hospitalists.

**DESIGN:**

Retrospective research design of 100 % Texas Medicare data using multilevel, multivariable models.

**SUBJECTS:**

131,710 hospitalized patients cared for by 1,099 hospitalists in 268 hospitals from 2006–2009.

**MAIN MEASURES:**

We calculated, for each hospitalist, adjusted for patient and disease factors (case mix), their patients' average length of stay, rate of discharge home or to skilled nursing facility (SNF) and rate of 30-day mortality, readmissions and emergency room (ER) visits.

**KEY RESULTS:**

In two-level models (admission and hospitalist), there was significant variation in average length of stay and discharge location among hospitalists, but very little variation in 30-day mortality, readmission or emergency room visit rates. There was stability over time (2008–2009 vs. 2006–2007) in hospitalist performance. In three-level models including admissions, hospitalists and hospitals, the variation among hospitalists was substantially reduced. For example, hospitals, hospitalists and case mix contributed 1.02 %, 0.75 % and 42.15 % of the total variance in 30-day mortality rates, respectively.

**CONCLUSIONS:**

There is significant variation among hospitalists in length of stay and discharge destination of their patients, but much of the variation is attributable to the hospitals where they practice. The very low variation among hospitalists in 30-day readmission rates suggests that hospitalists are not important contributors to variations in those rates among hospitals.

Hospitalists are physicians who specialize in the care of hospitalized patients. There are advantages and disadvantages to the “hospitalist” model. The potential advantages stem from greater efficiency and expertise from physicians concentrating just on inpatient care.^1^–^5^ The potential disadvantages derive from discontinuities in care: the unfamiliarity of the hospitalist with the patient and the communication errors that might occur during transitions from outpatient to inpatient, and vice versa, between different physicians.^6^–^16^ The negative impact of discontinuity on quality of care may be greater in the elderly.

We have used 5 % national Medicare data to describe the growth of hospitalists from 1996 through 2006,^17^,^18^ to evaluate the association of care by hospitalists with length of stay,^17^–^20^ to assess how the impact of hospitalists varies by patient and hospital characteristics,^18^ to examine how hospitalist care affects continuity of care,^21^–^23^ to describe the growing role of hospitalists in caring for surgical patients,^24^ and to describe the outcomes of hospitalist care.^19^,^20^,^23^,^25^ We found that hospitalist care was associated with shorter length of stay and lower hospital costs, but with higher medical costs post-discharge.^19^,^20^ In addition, patients receiving hospitalist care were less likely to be discharged to their homes and more likely to been seen in an emergency room (ER) in the 30 days after discharge.^19^,^20^

Variation in outcomes and quality of care that cannot be explained by illness severity, patient preference, or “unwarranted variation”, indicates an opportunity to decrease the cost or improve the effectiveness of healthcare.^26^–^29^ There is a substantial literature demonstrating that quality and outcomes of medical care vary among providers, and that this can be measured.^30^–^34^ However, to our knowledge, there have been no prior studies of variation in care among hospitalists. For example, are there significant, reproducible differences among hospitalists in the length of stay of their patients, in the percent of patients who are discharged home compared to a skilled nursing facility (SNF), or in 30-day readmission rates? What are the relative contributions of hospitalists, hospitals and patient case mix to readmission rates and other measures? In this report, we use 100 % Texas Medicare data to study 1,099 hospitalists practicing at 268 hospitals in Texas, and use multilevel models to study variation in length of stay and outcomes of care at the level of the individual hospitalist.

## METHODS

### Source of Data

Claims from the years 2005 to 2009 for 100 % of Texas Medicare beneficiaries were used, including Medicare beneficiary summary files, Medicare Provider Analysis and Review (MedPAR) files, Outpatient Standard Analytical Files (OutSAF), and Medicare Carrier files. Diagnosis related groups (DRG) associated information, including weights, Major Diagnostic Categories (MDC), and geometric mean length of stay, were obtained from Centers for Medicare & Medicaid Services (https://www.cms.gov/Medicare/Medicare-Fee-for-Service-Payment/AcuteInpatientPPS/index.html).

### Identification of Hospitalists

Hospitalists are defined as generalist physicians (general practitioner, family physician, internist or geriatrician) who had at least 100 evaluation-and-management (E&M) billings in a given year and generated at least 90 % of their total E&M billings in that year from inpatient services.^17^ Inpatient E&M billings were identified by Current Procedural Terminology (CPT) codes 99221-99223, 99231-99233 and 99251-99255. Outpatient E&M billings were identified by CPT codes 99201-99205, 99211-99215 and 99241-99245 from Carrier files.^17^ In sensitivity analyses, we varied the minimum number of E&M billings required for identification of hospitalists, and also the percentage of those bills from inpatient services. This had relatively small effect on the number of hospitalists identified. For example, raising the number of E&M charges to 200 from 100 decreased the number of hospitalists identified from 1,099 to 1,068, while reducing the percentage of E&M charges from 90 % to 75 % increased the number from 1,099 to 1,123.

### Establishment of the Study Cohort

This process is outlined in Table 1. From 2008 and 2009 MedPAR files, we started with all admissions and selected hospital admissions with a medical DRG from acute care hospitals in Texas. We excluded admissions with obstetric services, major trauma and intensive care unit (ICU) services. We excluded admissions with ICU stays, because the algorithm for identifying hospitalists cannot distinguish regular hospitalists from generalist physicians who are full-time intensive care physicians. We next identified admissions cared for by hospitalists. To identify these admissions, we first identified all the treating physicians for each hospitalization by linking inpatient E&M billings in the Carrier files to the admission record in MedPAR files. If all of the E&M billings by generalist physicians for a given admission were from hospitalists, the admission was considered an admission cared for by hospitalists. Among those admissions, we selected those in which one hospitalist was responsible for > 50 % of all hospitalist charges. For patients with more than one admission in a given year, we randomly selected one admission per patient per year, in order to avoid clustering at the patient level. In additional analyses with 30-day readmission rate as the outcome, we included all admissions for those patients with multiple admissions in a year. The results were almost identical. We further excluded patients who were enrolled in health maintenance organizations (HMOs) or did not have continuous Medicare Parts A and B coverage in the 12 months prior to the admission of interest, because such individuals may have incomplete information on covariates (such as comorbidity). This resulted in 138,761 admissions in the initial study cohort. From these, we selected admissions associated with a major hospitalist who cared for at least 30 admissions during the study period, leaving 131,710 admissions and 1,099 hospitalists. Hofer et al.^35^ has shown that provider-level performance measures have a reliability greater than 0.8 for a panel of 100 patients with an intraclass correlation coefficient (ICC) of 0.04. Depending on the particular outcome, additional selection criteria described in the Study Outcomes section were applied. We also built a cohort in the same manner from 2006 and 2007 MedPAR files, in order to study the consistency in performance of the hospitalists across two time periods.

**Table 1:** Selection of Cohorts of Admissions Cared for by Hospitalists. The Final Cohorts Differed Slightly Depending on the Analysis; For Example, We Excluded Deaths in Hospital when Measuring Post-Hospitalization Outcomes

	Admission Number (% of the last step)			
Admissions in 2008 and 2009 of TX beneficiaries from TX hospitals	1,928,143			
↓				
Short stay admissions only (no rehabilitation hospitals)	1,624,548 (84.3)			
↓				
Admissions with medical DRG only	1,142,137 (70.3)			
↓				
Exclude admissions with MDC 14 or 24	1,139,954 (99.8)			
↓				
Exclude admissions with ICU stay	764,777 (67.1)			
Admissions cared by generalists (any inpatient E&M from a generalist physician)	514,215 (67.2)			
↓				
Admissions where hospitalists are responsible for 100 % of E&M charges from generalist physicians	210,542 (40.9)			
↓				
Admissions with a major hospitalist (responsible for ≥ 50 % of all E&M billings from hospitalists)	190,077 (90.3)			
↓				
For patients with multiple admissions in one year, randomly select one admission	153,932 (81.0)			
↓				
Admissions from patients with complete Parts A & B and no HMO in the year before admission	138,761 (90.1)			
↓				
Further selection	Length of Stay	Discharge Home/SNF^*^	30-day Readmission^†^	30-day ER visit^†^
		107,901 (77.8)	116,228 (83.8)	115,928 (83.5)
↓				
Admissions with a major hospitalist who cared more than 30 admissions	131,710 (94.9)	99,522 (92.2)	108,547 (93.4)	108,226 (93.4)
	↓			
Exclude outliers with > 3 standard deviations	129,491 (98.3)			

*TX* Texas; *DRG* diagnosis related group; *MDG* Major Diagnostic Category; *ICU* intensive care unit; *E&M* evaluation and management; *HMO* health maintenance organization; *SNF* skilled nursing facility; *ER* emergency room

^*^Those discharged dead, or to another acute care hospital or admitted from a nursing facility were excluded

^†^Those discharged dead, or to another acute care hospital or dead without an event (readmission or ER visit) within 30 days were excluded

### Covariates

We categorized beneficiaries by age, gender and ethnicity using Medicare beneficiary summary files. We used the Medicaid indicator as a proxy of low income. Information on weekday vs. weekend admission, emergent admission, and DRG were obtained from MedPAR files. Elixhauser medical conditions were identified using the claims from MedPAR, Carrier and OutSAF files in the year prior to that of the admission of interest.^36^ We also assessed whether a patient had a primary care physician (PCP). A PCP was defined as a general practitioner, family physician, internist or geriatrician who saw the patient on three or more occasions in an outpatient setting (CPT E&M codes 99201-99205 and 99211-99215) in the prior year.^37^ Total hospitalizations and outpatient visits in the prior year were identified from MedPAR files and Carrier files, respectively.

### Study Outcomes

Hospital length of stay was obtained from MedPAR files. For each admission, we calculated a difference in length of stay by subtracting the geometric mean length of stay for that DRG obtained from the Center for Medicare and Medicaid Services from the actual length of stay. This measure intrinsically controls for case mix among hospitalists, because geometric mean length of stay differs for each DRG. We excluded outliers more than three standard deviations from the norm in order to approximate the normal distribution and analyze with a hierarchical general linear model, leaving 129,491 admissions, and 1,099 hospitalists.

Mortality within 30 days of admission was calculated from date of death in the Medicare beneficiary summary file. These analyses included all 131,710 admissions and 1,099 hospitalists in the cohort. We chose mortality within 30 days of admission rather than from discharge to avoid biases in different hospital length of stay among hospitalists. However, our analyses of 30-day post discharge mortality produced almost identical results.

We calculated the rate of admissions discharged home and the rate discharged to a Skilled Nursing Facility (SNF), obtained from MedPAR files. We excluded those who were discharged dead, transferred to another acute care hospital or had stayed in a nursing facility any time in the three months prior to the admission of interest, leaving 99,522 admissions and 990 hospitalists.

ER visits were identified by CPT E&M codes 99281-99285 and 99288 from Carrier files. To study readmissions and ER visits within 30 days of discharge, we excluded those who were discharged dead or transferred to another acute care hospital, or died in the 30 days post discharge without an event (readmission or ER visit), leaving 108,547 admissions and 1,019 hospitalists in the study cohort for 30-day readmission, and 108,226 admissions and 1,018 hospitalists for 30-day ER visits. Readmissions and ER visits were not mutually exclusive; i.e., most readmissions also had an ER visit.

### Statistical Analyses

Multilevel analyses were used to account for the clustering of patients within hospitalists and hospitalists within hospitals. For differences in length of stay, a hierarchical general linear model was used. For other outcomes, we used hierarchical generalized linear models with binomial distribution. The hospitalist-specific estimates were derived from two-level models adjusted with patient characteristics and then plotted by rank, and from three-level models including hospitals. Patient characteristics included age, race/ethnicity, gender, Medicaid eligibility, emergency admission, weekend admission, DRG weight, MDC, Elixhauser medical condition (29 individual indicators), number of hospitalizations, number of physician visits and having a PCP in the year prior to the admission of interest. For the model analyzing differences in length of stay, DRG weight was not adjusted because it was a within-DRG comparison. Because some hospitalists cared for admissions at more than one hospital in the three-level models, we assigned hospitalists to the hospital in which > 50 % of their E&M charges occurred, and excluded admissions by those hospitalists to other hospitals. All analyses were performed with SAS version 9.2 (SAS Inc., Cary, NC). The threshold models for the partitioned variances were performed with MLwiN version 2.02.^38^

## RESULTS

The final sample was 131,710 admissions cared for by 1,099 hospitalists. The median number of Medicare admissions cared for by each hospitalist was 98, and the 25^th^ and 75^th^ percentiles were 54 and 156. The characteristics of those admissions are summarized in Table 2. The average length of stay was 4.2 days. For patients admitted from home, 81.5 % were discharged back to their home, with the remainder going to a SNF, rehabilitation or other inpatient facilities. The 30-day readmission rate was 15.8 %; 19.8 % were seen in an ER within 30 days of discharge; and mortality within 30 days after admission was 7.7 %.

**Table 2:** Characteristics of Hospitalized Patients Cared for by Hospitalists

Patient Characteristic	N	%
Overall	131,710	100
Age		
≤ 65	23,522	17.9
66–70	17,493	13.3
71–75	19,460	14.8
76–80	21,794	16.5
81–85	22,537	17.1
> 85	26,904	20.4
Gender		
Male	52,631	40.0
Female	79,079	60.0
Race		
White	105,916	80.4
Black	15,152	11.5
Hispanic	8,262	6.3
Other	2,380	1.8
Major Diagnostic Category		
Nervous System	11,239	8.5
Eye	179	0.1
Ear, Nose, Mouth And Throat	1,356	1.0
Respiratory System	25,144	19.1
Circulatory System	23,262	17.7
Digestive System	15,861	12.0
Hepatobiliary System And Pancreas	3,662	2.8
Musculoskeletal System And Connective Tissue	6,238	4.7
Skin, Subcutaneous Tissue And Breast	5,272	4.0
Endocrine, Nutritional And Metabolic System	7,247	5.5
Kidney And Urinary Tract	14,203	10.8
Male Reproductive System	348	0.3
Female Reproductive System	251	0.2
Blood and Blood Forming Organs and Immunological Disorders	2,928	2.2
Myeloproliferative Diseases and Disorders and Poorly Differentiated Neoplasms	519	0.4
Infectious and Parasitic Diseases and Disorders	7,355	5.6
Mental Diseases and Disorders	3,324	2.5
Alcohol/Drug Use or Induced Mental Disorders	435	0.3
Injuries, Poison And Toxic Effect of Drugs	1,303	1.0
Burns	34	0.0
Factors Influencing Health Status	1,305	1.0
Human Immunodeficiency Virus Infection	245	0.2
Medicaid Eligible	40,483	30.7
Having a PCP in Prior Year	61,166	46.4
Emergency Admission	101,846	77.3
Weekend Admission	34,994	26.6
Elixhauser Comorbidity Score		
0	11,248	8.5
1 or 2	32,267	24.5
3 or 4	33,848	25.7
5 or 6	26,106	19.8
≥ 7	28,241	21.4
Mean (± SD) DRG Weight	1.0 ± 0.4	
Mean (± SD) Number of Hospitalizations in Prior Year	1.1 ± 1.7	
Mean (± SD) Number of Doctor Visits in Prior Year	9.8 ± 9.5	
Mean (± SD) Length of Stay in Days	4.2 ± 3.2	
30-Day Mortality	7.7	
% Discharged Home^*^	81.5	
% Discharged SNF^*^	11.0	
30-day Readmission Rate^†^	15.8	
30-day ER Visit Rate^‡^	19.8	

*DRG* diagnosis-related group; *SNF* skilled nursing facility; *ER* emergency room

^*^Those discharged dead, or to another acute care hospital, or admitted from a nursing facility were excluded, leaving 99,522 discharges. In addition to the 92.5 % of patients discharged either home or to a skilled nursing facility, other discharge destinations included inpatient rehabilitation (3.3 %), chronic care hospitals (1.7 %), hospice (1.1 %), left against medical advice (0.9 %) and other (0.5 %)

^†^Those discharged dead, or to another acute care hospital or dead without readmission within 30 days were excluded, leaving 116,228 discharges

^‡^Those discharged dead, or to another acute care hospital or dead without ER visit within 30 days were excluded, leaving 108,226 discharges

Our primary interest was variation in patient length of stay, discharge location and 30-day outcomes, at the level of each hospitalist. For this, we conducted a series of two-level models, controlling for the characteristics in Table 2.

**Figure 1.**
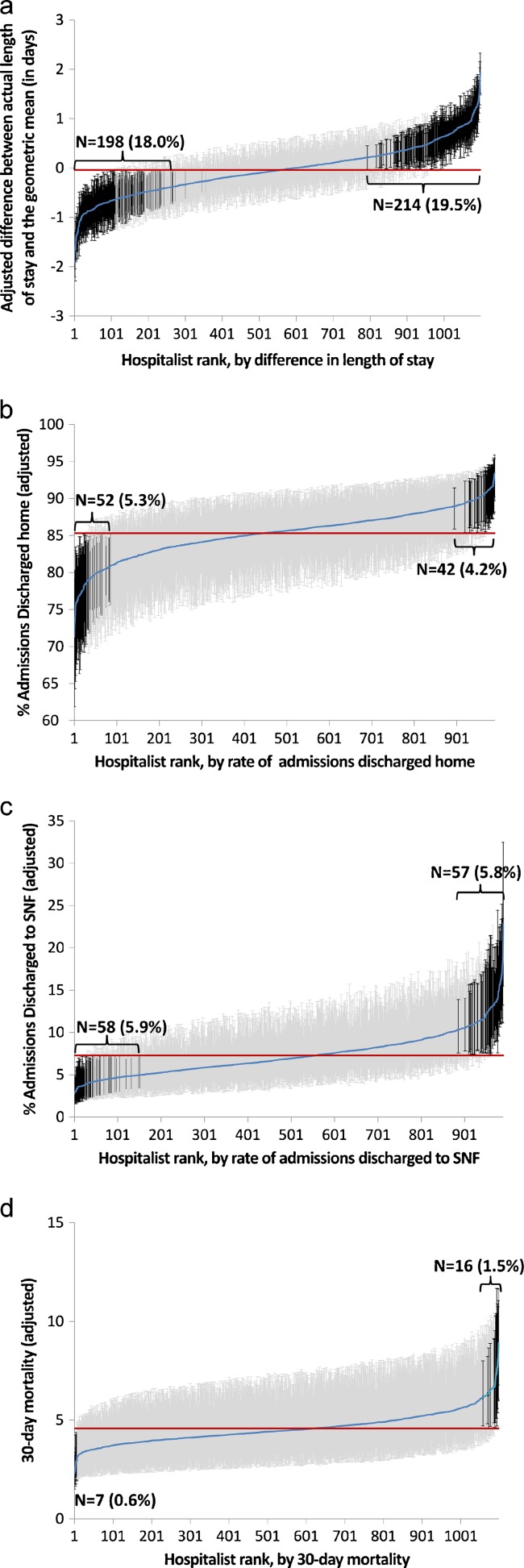
Differences in length of stay (**a**); rates of admissions discharged home (**b**); rate discharged to skilled nursing facility (**c**); and 30-day mortality rates (**d**) for Texas hospitalists, from lowest to highest. The differences or rates were estimated by 2-level analyses, adjusted with patient characteristics. The horizontal line represents the overall mean. Error bars represent 95 % confidence intervals of the estimate for the individual hospitalist. Black error bars represent hospitalists with significantly higher or lower estimates.

Figure 1a shows the variation in length of stay for each hospitalist. This is a cumulative distribution showing the mean value and 95 % confidence intervals for each hospitalist, derived from the two-level multivariable model. Dark vertical lines indicate hospitalists whose average length of stay is significantly different from the mean. The patients of 198 hospitalists (18 %) had significantly shorter lengths of stay, while the patients of 214 hospitalists (19 %) had significantly longer lengths of stay. A similar pattern is shown for the percent of patients discharged home (Fig. 1b) and discharged to a SNF (Fig. 1c), but with fewer hospitalists being significantly different from the mean. Very few hospitalists were significantly different from the mean in 30-day mortality (Fig. 1d). There were no significant differences among hospitalists in 30-day readmission rates. For 30-day ER visit rates, only one hospitalist was significantly higher and two significantly lower than the mean (data not shown).

We evaluated the stability of hospitalist performance over time by assessing the average adjusted length of stay for hospitalists in 2008–2009 compared to their length of stay in 2006–2007. We ranked the 633 hospitalists with > 30 admissions in both 2006–2007 and 2008–2009 by quintile of their average adjusted length of stay in 2008–2009 (Table 3). Of hospitalists in the first quintile in length of stay in 2008–2009, 50.8 % were in the first quintile and 25.4 % were in the second quintile in 2006–2007. For those in the fifth quintile in 2008–2009, 56.3 % were in the fifth quintile and 30.2 % in the fourth quintile in 2006–2007.

**Table 3:** Comparison of the Rank of 633 Hospitalists, by Quintile, in Adjusted Length of Stay of Their Patients, in 2006–2007 Versus 2008–2009

2006–2007 Quintiles	2008–2009 Quintiles				
	Q1 (*n* = 126)	Q2 (*n* = 127)	Q3 (*n* = 127)	Q4 (*n* = 127)	Q5 (*n* = 126)
Q1	50.8 %	22.0 %	20.5 %	5.5 %	0.8 %
Q2	25.4 %	31.5 %	25.2 %	13.4 %	5.6 %
Q3	13.5 %	26.8 %	22.0 %	29.1 %	7.1 %
Q4	7.1 %	10.2 %	24.4 %	29.1 %	30.2 %
Q5	3.2 %	9.4 %	7.9 %	22.8 %	56.3 %

The numbers given are the percent of hospitalists in a given quintile of adjusted length of stay in 2008–2009, who were in that same quintile, or another quintile, in 2006–2007. The Wilcoxon signed rank test found no significant difference in the distribution of hospitalists among the quintiles in the two time periods (*p* = 0.80)

The two-level models described above do not account for the fact that hospitalists cluster within hospitals. Therefore, to distinguish variation at the hospitalist level from variation among hospitals, we constructed three-level models examining the contribution of patient, hospitalist and hospital to variations in length of stay, discharge destination and 30-day outcomes. Table 4 presents the proportion of the variation (ICC) at the hospitalist and hospital level for each of these measures. Also shown is the partitioned variance, which is the percentage of total variance contributed by hospitals, hospitalists and measurable patient factors (case mix). For length of stay, hospitals and hospitalists contributed roughly equally to the variation, while for discharge destination (home or SNF), the hospital contribution was larger than that of hospitalists. The variance at the hospital and hospitalist level in 30-day readmission, 30-day ER visit rates and mortality were small, but significantly greater than 0. Similarly, the ICCs for these outcomes were small. For the 30-day outcomes, the contribution of patient-level factors was one to two orders of magnitude higher than that of hospital- and hospitalist-level factors.

**Table 4:** Variation Contributed by Hospital-Level and Hospitalist-Level Variables to Outcomes in Three-Level Models

	Difference in LOS	Discharged Home	Discharged SNF	30-day Readmission	30-day ER visit	30-day Mortality
	ICC (%)*					
Hospital	3.33	3.10	6.27	0.27	0.47	1.82
Hospitalist	3.48	1.37	2.31	0.41	0.35	1.52
	Partitioned Variance^†^ (%)					
Hospital	2.94	1.78	3.56	0.09	0.37	1.02
Hospitalist	2.60	0.73	1.00	0.18	0.12	0.75
Case Mix	11.54	37.23	39.74	24.03	22.70	42.15

*LOS* length of stay; *SNF* skilled nursing facility; *ER* emergency room; *ICC* intraclass correlation coefficient

For the model on LOS, there were 203 hospitals, 1064 hospitalists, and 113,289 admissions. The numbers in each category varied slightly between the six models (see Fig. 1)

* The percentage of variation attributable to the hospitalist and hospital was calculated from 3-level null models. All values presented are significantly different from zero (*p* < 0.05)

^†^The models were adjusted for patient characteristics, including age, race, sex, Medicaid eligibility, emergency admission, weekend admission, diagnosis related group (DRG) weights, major diagnostic category, Elixhauser comorbidity (29 indicators), number of hospitalizations, number of doctor visits and whether the patient had an identifiable primary care physician in the year before admission. DRG weight was not adjusted when modeling the difference in LOS, because differences in LOS involve comparisons within the same DRG. The variance was partitioned using a threshold model so as to present the percentages of total variance contributed by hospital-level, hospitalist-level and patient-level characteristics (case mix). Results are presented as the percentage of total variance attributable to the indicated factor. The denominator is total variance, composed of the variance attributable to hospitals, hospitalists, measured patient characteristics and that attributable to unexplained patient characteristics plus error. All values shown are significantly different from zero (*p* < 0.05)

In all the analyses above, if a patient had multiple hospitalizations in a given year, we selected one at random to avoid clustering at the patient level. However, this method would tend to lower estimates of the rehospitalization rate. Accordingly, in supplemental analyses we included all admissions. This had almost no effect on the estimates of the ICC for readmission.

## DISCUSSION

The purpose of this study was to assess the extent of variation in the care provided by hospitalists by measures of their practice: average adjusted length of stay, discharge destination, 30-day mortality, and rates of readmission and ER visits within 30 days of discharge. We found significant variation in length of stay and discharge destination among hospitalists, a variation that was stable over time. However, much of the variation among hospitalists was because of clustering of hospitalists within hospitals. We found little to no variation among hospitalists in 30-day mortality, rehospitalization or ER visit rates, either in the two-level or three-level models.

The variations in lengths of stay and discharge destination, between hospitalists and between hospitals, suggest underlying variations in hospitalist practice styles and hospital-based systems of care. The relative lack of variation at the hospitalist level in 30-day outcomes is not likely due to insufficient power, given the large number of hospitalists and substantial number of admissions per hospitalist. This suggests that the hospitalist practice styles that lead to variations in length of stay and discharge destination do not have a noticeable impact on mortality, readmission rates or ER visit rates. Several prior studies have also suggested a weak link between care in hospital and readmission rates.^39^–^41^ First, practice styles leading to better performance on measures of hospital discharge planning are not associated with significant improvement in readmission rates.^39^ Second, regional baseline admission rates (a measure of primary care practice styles and systems) have a much greater influence on readmission rates than patient or hospital factors.^40^ Third, most interventions (largely impacting hospital-based practice styles or systems) have failed to substantially reduce the risk of readmission.^41^

Hospitals are being held accountable for readmissions of patients they discharge.^42^ Hospitals are likely to shift some of this accountability to their hospitalists. Our findings suggest that this shift may be misguided. In the three-level model apportioning variance in readmission rates among hospitals, hospitalists and measurable patient characteristics (case mix), the percent of total variance from hospitals (0.09 %) and hospitalists (0.18 %) was more than two orders of magnitude lower than that attributable to case mix (24.03 %).

Our study has limitations. It is an observational study and susceptible to bias and confounding. We studied patients with fee-for-service Medicare who received care in a single large state in the USA over a two-year period. It is possible that our results may not apply to a younger population, those in other states, or during a different time period. In particular, there are substantial variations in hospital readmission rates among different regions of the USA.^39^,^40^ This variation would be missed in the current analyses. We excluded patients with ICU stays in this study, so our results do not apply to critically ill patients. We focused on variation among hospitalists, but did not examine the impact of characteristics of individual hospitalists (years of experience, training, etc.) on that variation. We also did not look at continuity of care, i.e., whether the patient was cared for by one, two or several hospitalist while hospitalized.

As hospitalist care continues to increase in prevalence in the USA, so does the ability of hospitalists to impact the cost and quality of hospital care.^17^ Our study suggest a potential opportunity for hospitalists to further impact the cost of care by decreasing variability in their clinical practice that leads to the variation in length of stay and discharge location. Our study also suggests that an approach of making hospitalists accountable for decreasing the cost of care related to readmissions may be flawed, and could lead to unintended negative consequences.^43^

## References

[1] Wachter RM, Goldman L (1996). The emerging role of “hospitalists” in the American health care system. N Engl J Med.

[2] Bryant DC (1999). Hospitalists and ‘officists’ preparing for the future of general internal medicine. J Gen Intern Med..

[3] Coffman J, Rundall TG (2005). The impact of hospitalists on the cost and quality of inpatient care in the United States: a research synthesis. Med Care Res Rev.

[4] Wachter RM (2006). Reflections: the hospitalist movement a decade later. J Hospital Med.

[5] Wachter RM (2008). The state of hospital medicine in 2008. Med Clin North Am.

[6] Hinami K, Farnan JM, Meltzer DO, Arora VM (2009). Understanding communication during hospitalist service changes: a mixed methods study. J Hosp Med.

[7] Kripalani S, LeFevre F, Phillips CO, Williams MV, Basaviah P, Baker DW (2007). Deficits in communication and information transfer between hospital-based and primary care physicians: implications for patient safety and continuity of care. JAMA.

[8] Pham HH, Grossman JM, Cohen G, Bodenheimer T (2008). Hospitalists and care transitions: the divorce of inpatient and outpatient care. Health Aff (Millwood).

[9] Meltzer D (2001). Hospitalists and the doctor-patient relationship. J Legal Stud.

[10] Coleman EA, Berenson RA (2004). Lost in transition: challenges and opportunities for improving the quality of transitional care. Ann Intern Med.

[11] Roy CL, Poon EG, Karson AS (2005). Patient safety concerns arising from test results that return after hospital discharge. Ann Intern Med.

[12] Moore C, Wisnivesky J, Williams S, McGinn T (2003). Medical errors related to discontinuity of care from an inpatient to an outpatient setting. J Gen Intern Med.

[13] Coleman EA, Min SJ, Chomiak A, Kramer AM (2004). Posthospital care transitions: patterns, complications and risk identification. Health Serv Res.

[14] Frustrations with hospitalist care: need to improve transitions and communication. Ann Intern Med. 2010;152:469. 10.7326/0003-4819-152-7-201004060-00013 20368654

[15] Adult MJ (2010). The relationship between hospitalist and primary care physicians. Ann Intern Med.

[16] Snow V, Beck D, Budnitz T (2009). Transitions of Care Consensus policy statement: American College of Physicians, Society of General Internal Medicine, Society of Hospital Medicine, American Geriatrics Society, American College of Emergency Physicians, and Society of Academic Emergency Medicine. J Gen Intern Med.

[17] Kuo YF, Sharma G, Freeman JL, Goodwin JS (2009). Growth in the care of older patients by hospitalists in the United States. New Eng J Med.

[18] Kuo YF, Goodwin JS (2010). Effect of hospitalist on length of stay in the Medicare population: variation according to hospital and patient characteristics. J Am Geriatr Soc.

[19] Howrey B, Kuo YF, Goodwin JS (2011). Association of care by hospitalists on discharge destination and 30-day outcomes following acute ischemic stroke. Medical Care.

[20] Kuo YF, Goodwin JS (2011). Association of hospitalist care with medical utilization after discharge: evidence or cost shifting. Ann Intern Med.

[21] Fletcher KE, Sharma G, Zhang DD, Kuo YF, Goodwin JS (2011). Trends in inpatient continuity of care for a cohort of Medicare patients 1996–2006. J Hosp Med.

[22] Sharma G, Fletcher KE, Zhang DD, Kuo Y-F, Freeman JL, Goodwin JS (2009). Continuity of outpatient and inpatient care for hospitalized older adults. JAMA.

[23] Sharma G, Kuo YF, Freeman JL, Zhang DD, Goodwin JS (2010). Outpatient follow-up with a usual care provider and 30-day emergency room visit and readmission in patients hospitalized for chronic obstructive pulmonary disease. Arch Inter Med.

[24] Sharma G, Kuo YF, Freeman J, Goodwin JS (2010). Comanagement of hospitalized surgical patients by medicine physicians in the United States. Arch Intern Med.

[25] Sharma G, Freeman JL, Zhang DD, Goodwin JS (2009). Continuity of care and Intensive Care Unit use at the end of life. Arch Intern Med.

[26] Wennberg JE (2002). Unwarranted variations in healthcare delivery: implications for academic medical centres. BMJ.

[27] **Wennberg DE, Wennberg JE.** Addressing variations: Is there hope for the future? Health Aff (Millwood). 2003;Suppl Web Exclusives:W3-614-7. 10.1377/hlthaff.w3.614 15506164

[28] Mercuri M, Gafni A (2011). Medical practice variations: what the literature tells us (or does not) about what are warranted and unwarranted variations. J Eval Clin Pract.

[29] Wennberg JE (2011). Time to tackle unwarranted variations in practice. BMJ.

[30] Schroeder SA, Schliftman BA, Piemme TE (1974). Variation among physicians in use of laboratory tests: relation to quality of care. Med Care.

[31] Scholle SH, Roski J, Adams JL (2008). Benchmarking physician performance: reliability of individual and composite measures. Am J Manag Care.

[32] NBCH: National Business Coalition on Health. Value-based Purchasing Guide. Chapter 2: Physician Performance Measurement and Reporting. Available at: http://www.nbch.org/VBPGuide. Accessed November 30, 2011.

[33] Westert GP, Nieboer AP, Groenewegen PP (1993). Variation in duration of hospital stay between hospitals and between doctors within hospitals. Social Sci Med.

[34] de Jong JD, Westert GP, Lagoe R, Groenewegen PP (2006). Variation in hospital length of stay: do physicians adapt their length of stay decisions to what is usual in the hospital where they work?. Health Serv Res.

[35] Hofer TP, Hayward RA, Greenfield S, Wagner EH, Kaplan SH, Manning WG (1999). The unreliability of individual physician “report cards” for assessing the costs and quality of care of a chronic disease. JAMA.

[36] Elixhauser A, Steiner C, Harris DR, Coffey RM (1998). Comorbidity measures for use with administrative data. Med Care.

[37] Shah BR, Hux JE, Laupacis A, Zinman B, Cauch-Dudek K, Booth GL (2007). Administrative data algorithms can describe ambulatory physician utilization. Health Serv Res.

[38] **Rasbash J, Charlton C, Browne WJ, Healy M, Cameron B.** MLwiN Version 2.02. Centre for Multilevel Modelling, University of Bristol; 2005.

[39] Jha AK, Orav EJ, Epstein AM (2009). Public reporting of discharge planning and rates of readmissions. New Engl J Med.

[40] Epstein AM, Jha AK, Orav EJ (2011). The relationship between hospital admission rates and rehospitalizations. New Engl J Med.

[41] Hansen LO, Young RS, Hinami K, Leung A, Williams MV (2011). Interventions to reduce 30-day rehospitalization: a systematic review. Ann Int Med.

[42] Medicare Program, Hospital Inpatient Prospective Payment Systems for Acute Care Hospitals and the Long-Term Care Hospital Prospective Payment System and FY 2012 Rates, Hospitals’ FTE Resident Caps for Graduate Medical Education Payment (2011). Fed Regist Rules Regul.

[43] Axon RN, Williams MV (2011). Hospital readmission as an accountability measure. JAMA.

